# Co-Infections and Sero-Prevalence of HIV, Syphilis, Hepatitis B and C Infections in Sexually Transmitted Infections Clinic Attendees of Tertiary Care Hospital in North India

**Published:** 2016-08-04

**Authors:** Sonali Bhattar, Prabhav Aggarwal, Satyendra Kumar Sahani, Preena Bhalla

**Affiliations:** ^a^ Department of Microbiology, Maulana Azad Medical College and Associated Lok Nayak Hospital, New Delhi, India; ^b^ Department of Dermatology, Maulana Azad Medical College and Associated Lok Nayak Hospital, New Delhi, India

**Keywords:** HIV, Syphilis, Hepatitis B, Hepatitis C

## Abstract

**Background:** HIV, syphilis, hepatitis B and C (HBV & HCV) infections modify the epidemiology and presentation of each other. This study aimed to estimate the seroprevalence of these infections and their co-infections in sexually transmitted infections (STI) clinic attendees in New Delhi, India.

**Methods:** A retrospective study including 220 patients was conducted during May 2014 through December 2014. Serodiagnosis of HIV was performed as per Strategy III of NACO guidelines; syphilis by VDRL followed by TPHA; HBV and HCV by rapid immuno-chromatographic test followed by ELISA.

**Results:** Male subjects were slightly more in number as compared to females (56.36% vs. 43.63%). Twelve (5.45%), 14 (6.36%), three (1.36 %) and one (0.45%) were reactive for HIV, VDRL, HBV and HCV, respectively. Three were both HIV and syphilis positive and one was both HIV and HBV positive; no co-infections of HBV/HCV, HIV/HBV/HCV and HIV/HBV/HCV/syphilis coexisted.

**Conclusions:** High prevalence of HIV, HBV, HCV and syphilis in STI clinic attendees mandate routine screening to detect co-infections and follow prompt therapy in order to minimize their sequelae.

## Introduction

Sexually transmitted infections (STIs) are important health related issues both globally as well as in India. Around three-fourths of the estimated 340 million annual new cases of curable STIs occur in the developing countries, and STIs account for 17% economic losses because of ill health^1^. In India, approximately 6% of the adult population suffers from STIs/RTIs^2^.HIV, syphilis, hepatitis B virus (HBV) and hepatitis C virus (HCV) are four common infections associated with systemic manifestations and grave consequences that share common routes of transmission, the most common being sexual route.

Other than sexual route, other parenteral routes such as sharing of needles, needle-stick injury, blood transfusion, organ transplantation, etc. are also important, more so for hepatitis C. The burden of these infections is immense. It is estimated that in 2015, there were 36.7 million people living with HIV^3^. In India, 0.26% of the general adult population is infected with HIV^4^. “More than 2,000 million people alive today have been infected with HBV at some time in their lives^ ”5^. Likewise, an estimated 2%–3% of the world's population is living with HCV infection, and 350,000 die annually of HCV-related conditions, including cirrhosis and liver cancer^6^. Lastly, approximately 6 million new cases of syphilis are reported annually^7^.

Individually, each of these four infections is capable of causing high morbidity and mortality; however, their co-infections with each other alter the disease course for worse. While syphilis facilitates HIV transmission, HIV/HCV and HIV/HBV co-infections facilitate disease progression to liver failure, cirrhosis or death^8^.Therefore, a holistic approach for early diagnosis and treatment is a necessity.

STI clinic attendees are screened for HIV and syphilis routinely; however, screening for other parenteral co-infections such as HBV and HCV is usually not done in present healthcare systems. Hence, our study aimed to estimate the seroprevalence of HIV, syphilis, HBV, HCV and their co-infections amongst high risk STI clinic attendees at a tertiary care center of North India.

## Methods

### Study Design

A retrospective study was conducted at the Department of Microbiology, Maulana Azad Medical College and associated 1600-bedded Lok Nayak Hospital, New Delhi, India. of a tertiary care center in north India. The data from May 2014 through December 2014 was retrieved from hospital records. All the first time attendees in the STI clinic presenting during the study period, irrespective of signs and symptoms were included in the study; and their relevant clinical and socio-demographic data were collected. All attendees were screened for syphilis, HIV, hepatitis B and hepatitis C infection, after taking requisite consent and ensuring that their identities would be kept confidential. Repeat visitors were excluded from the study.

### Microbiological Testing Methodology

Diagnosis of HIV infection was performed strictly as per the Strategy III of NACO guidelines at the HIV Laboratory in the Department of Microbiology, Maulana Azad Medical College, New Delhi, India^9^. Three ml of blood sample was collected in plain Vacutainer® vial (Becton, Dickinson and Company, USA) and allowed to clot. Serum was separated by centrifuging at 2,000-2,500 rpm for 15 min. Three different rapid tests were used to detect HIV-1 and HIV-2 antibodies (COMBAIDS (Span Diagnostics Ltd.), Retrocheck HIV (Qualpro Diagnostics), and Tri-line (Rapid Diagnostics) as per the manufacturer’s instructions.

For diagnosis of syphilis, all samples were tested initially by Venereal Disease Research Laboratory test (VDRL) test as per the standard protocol (Serologist to Govt. of India, Kolkata, India). VDRL reactive specimens were subjected to quantitative VDRL test with successive two fold dilutions of the serum in 0.9% saline. *Treponema Pallidum* Hemagglutination (TPHA) test (Plasmatec Laboratory Products Ltd.) was done for samples that were reactive or weakly reactive by VDRL test.

The sera samples were also tested for hepatitis B surface antigen (SD BIOLINE HBsAg, Standard Diagnostics, Inc.) and anti-HCV antibodies (SD BIOLINE HCV, Standard Diagnostics, Inc.); both of which were further confirmed by ELISA.

### Statistical Analysis

Statistical analysis was performed using Epi Info software version 7.1.14. Chi-square analysis with Yate’s correction was used for discrete variables and *P* <0.05 were considered statistically significant. The descriptive data is presented as mean **±** standard deviation (SD) and percentages. 95% Confidence Intervals (95% CI) were calculated wherever relevant.

## Results

The study involved a total of 220 subjects; including 124 (56.4%) males and 96 (43.6%) females. The age of the attendees ranged from 15 to 63 yr. Overall the mean age was 30.75 ± 9.67 yr; with male and female having mean age of 32.13 ± 10.33 yr and 29.01 ± 8.50 yr, respectively. Majority of study subjects were illiterate. One hundred and seventy-nine (81.4%) were married and 38 (17.3%) were single. The socio-demographic profile of all the participants is shown in Table 1.

**Table 1:** Socio-demographic profile of the study population (n=220)

**Variables**	**Numbers**	**Percentage**
**Age group (yr)**		
<20	16	7.3
20-29	97	44.1
30-44	85	38.6
≥45	22	10.0
**Gender**		
Male	124	56.4
Female	96	43.6
**Education**		
Illiterate	98	44.6
Primary school	77	35.0
Secondary school	25	11.4
College and above	20	9.1
**Occupation**		
Daily wages	112	50.9
House wife and Unemployed	65	29.6
Business	9	4.1
Salaried	21	9.6
Student	13	5.9
**Marital status**		
Married	179	81.4
Single	38	17.3
Divorced	1	0.5
Widowed	2	0.9

Out of 220 STI clinic attendees, a total of 12 (5.5%) attendees turned out to be HIV positive. Fourteen (6.4%) individuals were VDRL and TPHA reactive, with five persons having VDRL titers more than 1:8. Statistically significant differences were found in syphilis seroprevalence between males and females (*P*=0.044). Three attendees (1.4 %) were HBV positive, and only one (0.5%) was reactive for HCV antibodies. Three (two males and one female) out of 220 study participants were sero-reactive for both HIV and syphilis with co-prevalence rate of 1.4%. One male was sero-reactive for both HIV and HBsAg and 1 was reactive for HIV, HBsAg and syphilis. No co-infection of HBV/HCV, HIV/HBV/HCV and HIV/HBV/HCV/syphilis were observed (Table 2).

**Table 2:** Sero-prevalence of HIV, syphilis, Hepatitis B and Hepatitis C in STD clinic attendees

**Variables**	**Male (n=124)**	**Female (n=96)**	**Total (n=220)**	***P*** ** value**
Overall sero-prevalence			
HIV	9 (7.3)	3 (3.1)	12	0.299
Syphilis	12 (9.7)	2 (2.0)	14	0.044
Hepatitis B	2 (1.6)	1 (1.0)	3	0.717
Hepatitis C	1 (0.8)	0 (0.0)	1	0.378
HIV and Syphilis	2 (1.6)	1 (1.0)	3	0.717
HIV and HBV	1 (0.8)	0 (0.0)	1	0.378
HIV, HBV and Syphilis	1 (0.8)	0 (0.0)	1	0.378
No co-infections				
HIV	5 (4.0)	2 (2.1)	7	0.668
Syphilis	10 (8.1)	1 (1.0)	11	0.396
Hepatitis B	1 (0.8)	0 (0.0)	1	0.378
Hepatitis C	1 (0.8)	0 (0.0)	1	0.378

Relationship between HIV, syphilis and hepatitis B co-infections is depicted in Table 3. There was a statistically significant difference in syphilis infection between the HIV reactive and non-reactive STI attendees (*P* = 0.032). Similarly, difference in HBV infection between the HIV reactive and non-reactive was statistically significant (*P* = 0.001)

**Table 3:** Sero-prevalence of Syphilis and Hepatitis B infection in HIV reactive and non-reactive individuals

**Variables**	**HIV reactive (n=12)**	**HIV non-reactive (n=208)**	**Total (n=220)**	***P*** ** value**
VDRL reactive	3 (25.0)	11 (5.3)	14	0.032
VDRL non-reactive	9 (75.0)	197 (94.7)	206	0.032
HBsAg positive	2 (16.7)	1 (0.5)	3	0.001
HBsAg negative	10 (83.3)	207 (99.5)	217	0.001

## Discussion

STIs remain a public health problem of major significance in most parts of the world^10,11^.The prevalence of STIs is high in many countries, including India. However, the exact figures are rarely available, as various studies quote highly variable prevalence rates^12^. Most STIs may present with genital lesions which are generally easy to identify and diagnose. However, other sexually transmissible infections may not show any obvious genital lesion making the clinical diagnosis rather difficult. Among the four infections discussed in the present study, only primary syphilis transiently leads to genital manifestations. These four infections (HIV, HBV, HCV and syphilis) all may enter long latent and/or carrier stage during with they cannot detected clinically.

A failure to diagnose and treat them at an early stage results in serious complications and sequelae such as infertility, fetal wastage, ectopic pregnancy, liver cirrhosis, carcinoma, acquisition of a plethora of opportunistic infections and eventual death^13^. Thus it becomes essential to have knowledge regarding their prevalence in the population.

In our study, the majority of the study subjects were in the most sexually active age group of 20-29 yr, as in Northern India^13^. The present study population consisted mostly of married clients with low level of education which correlated well with another Indian study^12^.

The overall seroprevalence of HIV (i.e. 5.45%) among STI clinic attendees, recorded in this study is much higher than the national HIV prevalence of 0.26% general population ^4^; although our finding is consistent with the finding of Jindal et al. in STI attendees^14^.This is understandable as patients presenting to STI clinics are likely to be belonging to high risk category for HIV infection.

Overall seropositivity for syphilis was 6.36%; with males and females having seroprevalence of 9.67% and 2.08%, respectively. In another study, the seropositivity was 8.2%, with higher prevalence in females (females = 8.9% vs. males = 7.7%)^15^. However, similar to our study, in Iran reported higher prevalence of syphilis in men as compared to women.^16^ In our study, seroprevalence of HBV was 1.36% which is lower than previous reports ,^18^ stating a prevalence rate of 3.6% and 3%, respectively. The prevalence of seropositivity of HCV antibody in the present study was 0.45%, while in other studies it ranges from 0.8-2.6%^14,16^.

The co-infection of HIV/ syphilis and HIV/ HBV was 1.36% and 0.45% respectively, similar to another study where these co-infections were present in 1% and 0.2% study population, respectively^13^. In another study, co-infection of HIV/ HBV reported as 2%^11^. Keramat et al. reported co-infection of the viral infections in 4.5 % (17 cases) including 13 cases of HIV and HCV infections, 3 cases of HCV and HBV infections and 1 case with HIV and HBV infections^19^. Although the percentage of patients with co-infections is lower, the combination of these infections is a dangerous co-existence and may have a detrimental effect on the patient and the treatment outcome^20,21^. Further studies are required to determine the variables influencing these co-infections such as a study that determined the association between *M. tuberculosis*/HIV co-infection with imprisonment previous TB infection, isoniazid preventive therapy, anti-retroviral therapy and CD4 count <350 cells/mm3^22^.

A timely diagnosis of these co-infections can minimize downstream adverse health effects, offset rapid disease progression, encourage cure and, most importantly, reduce transmission to partners and children. These will cumulatively decelerate co-infection epidemics.

Our study was limited by the fact that it was performed in a single center during specific period. Further studies should aim to be prospective and multi-centric to extend the knowledge gained through the present study. The choice of diagnostic tests in the present study was based on standard protocol; use of newer diagnostic methods may reveal higher prevalence of co-infections^23^.

## Conclusions

The prevalence of HIV, syphilis, HBV and HCV is much higher in STI clinic attendees as compared to general population. Thus, all STI clinic attendees must be screened for these sexually and parenterally transmissible infections for their prompt treatment and prevention of sequelae. There is a need to support an approach of targeted screening of these infections, into the existing STI prevention and treatment services.

## Acknowledgments

The authors would like to thank the technical assistance provided by Ms. Seema Malhotra and Ms. Shyny.

## Conflict of interest statement

None.

## Highlights

- Married, young males were most frequent visitor to STI clinic.
- Prevalence of HIV infection in STI Clinic attendees found to be much higher than general population.
- HBV and HCV infection frequently present as co-infection with HIV/syphilis.
- Routine HBV and HCV testing is essential to prevent sequelae.
